# Supplementary material for: Flexible time-restricted eating combined with exercise in a free-living setting for middle-aged women with overweight/obesity: a randomized controlled trial
Source: Nat Commun. 2025 Nov 27;16:10659. doi: 10.1038/s41467-025-65678-z (PMC12660762; doi:10.1038/s41467-025-65678-z)
Supplement: Supplementary file 1 — Supplementary Information [file 41467_2025_65678_MOESM1_ESM.pdf]

Supplementary Table S1. Subgroup Analysis by Menopausal Status: Intention-to-Treat Population (N=104)

Table S1. Subgroup Analysis by Menopausal Status: Intention-to-Treat Population (N=104)

**Outcome**	**Interaction effect**	**Menopausal status**	**Group**	**Postintervention**					
				**Compared with CON**			**Compared with flexTRE+EX**		
				**Adjusted mean difference**	**99%CI**	**P value**	**Adjusted mean difference**	**99%CI**	**P value**
Fat mass kg	P<0.001	Premenopausal	flexTRE	-1.55	-2.88 to -0.22	**0.003***	1.49	-0.12 to 3.10	**0.017***
			EX	-1.09	-2.59 to 0.40	0.059	1.95	0.25 to 3.65	**0.003***
			flexTRE+EX	-3.04	-4.70 to -1.39	**<0.001***			
		Perimenopausal	flexTRE	-0.41	-2.37 to 1.56	0.596	1.85	0.16 to 3.55	**0.005***
			EX	-4.27	-5.99 to -2.54	**<0.001***	-2.01	-2.82 to -1.20	**<0.001***
			flexTRE+EX	-2.26	-3.70 to -0.82	**<0.001***			
		Postmenopausal	flexTRE	-1.67	-3.75 to 0.42	0.040	1.03	-1.07 to 3.12	0.207
			EX	-0.11	-1.60 to 1.38	0.849	2.58	1.05 to 4.11	**<0.001***
			flexTRE+EX	-2.69	-4.65 to -0.73	**<0.001***			
Body mass kg	P<0.001	Premenopausal	flexTRE	-1.64	-2.88 to -0.40	**<0.001***	1.84	0.32 to 3.36	**0.002***
			EX	-1.58	-2.91 to -0.26	**0.002***	1.90	0.31 to 3.49	**0.002***
			flexTRE+EX	-3.48	-5.07 to -1.90	**<0.001***			
		Perimenopausal	flexTRE	-1.01	-2.92 to 0.90	0.173	1.37	-0.29 to 3.02	0.034
			EX	-4.28	-5.90 to -2.65	**<0.001***	-1.90	-2.90 to -0.90	**<0.001***
			flexTRE+EX	-2.38	-3.87 to -0.89	**<0.001***			
		Postmenopausal	flexTRE	-2.19	-3.35 to -1.03	**<0.001***	1.29	-0.51 to 3.10	0.064
			EX	-0.68	-1.71 to 0.35	0.089	2.80	1.07 to 4.54	**<0.001***
			flexTRE+EX	-3.48	-5.25 to -1.72	**<0.001***			
Body mass index kg/m^2	P<0.001	Premenopausal	flexTRE	-0.77	-1.20 to -0.34	**<0.001***	0.80	0.12 to 1.47	**0.002***
			EX	-0.59	-1.12 to -0.06	**0.004***	0.98	0.23 to 1.73	**<0.001***
			flexTRE+EX	-1.57	-2.22 to -0.91	**<0.001***			
		Perimenopausal	flexTRE	-0.60	-1.09 to -0.10	**0.002***	0.22	-0.59 to 1.02	0.488
			EX	-1.17	-1.58 to -0.76	**<0.001***	-0.36	-1.10 to 0.38	0.216
			flexTRE+EX	-0.81	-1.59 to -0.03	**0.008***			
		Postmenopausal	flexTRE	-1.12	-1.68 to -0.57	**<0.001***	0.22	-0.52 to 0.97	0.436
			EX	-0.36	-0.86 to 0.15	0.067	0.99	0.28 to 1.70	**<0.001***
			flexTRE+EX	-1.35	-2.12 to -0.57	**<0.001***			

Body fat percentage %	P<0.001	Premenopausal	flexTRE	-1.41	-2.68 to -0.15	**0.004***	1.31	-0.41 to 3.03	0.050
			EX	-0.95	-1.96 to 0.05	**0.014***	1.77	0.29 to 3.25	**0.002***
			flexTRE+EX	-2.72	-4.02 to -1.43	**<0.001***			
		Perimenopausal	flexTRE	-0.45	-2.19 to 1.28	0.50	1.37	-0.45 to 3.19	0.052
			EX	-2.49	-3.66 to -1.33	**<0.001***	-0.67	-1.85 to 0.52	0.148
			flexTRE+EX	-1.83	-3.34 to -0.32	**0.002***			
		Postmenopausal	flexTRE	-1.27	-2.92 to 0.38	0.048	0.60	-1.06 to 2.27	0.350
			EX	-0.10	-1.27 to 1.07	0.827	1.77	0.56 to 2.98	**<0.001***
			flexTRE+EX	-1.87	-3.45 to -0.29	**0.002***			
Insulin mU/L	P<0.001	Premenopausal	flexTRE	1.10	-1.81 to 4.02	0.331	3.22	0.58 to 5.86	**0.002***
			EX	0.44	-3.71 to 4.59	0.785	2.55	-1.41 to 6.52	0.097
			flexTRE+EX	-2.11	-4.98 to 0.75	0.057			
		Perimenopausal	flexTRE	3.02	-0.52 to 6.55	0.028	1.35	-2.53 to 5.23	0.370
			EX	7.54	5.60 to 9.47	**<0.001***	5.31	1.78 to 8.84	**<0.001***
			flexTRE+EX	1.66	-0.56 to 3.88	0.053			
		Postmenopausal	flexTRE	1.73	-3.37 to 6.83	0.382	4.22	-0.76 to 9.21	0.029
			EX	0.37	-3.75 to 4.48	0.819	2.86	-0.97 to 6.69	0.054
			flexTRE+EX	-2.49	-7.46 to 2.48	0.196			
HOMA-IR	P=0.003	Premenopausal	flexTRE	0.17	-0.54 to 0.89	0.532	0.74	0.13 to 1.36	**0.002***
			EX	0.13	-0.85 to 1.10	0.738	0.70	-0.23 to 1.62	0.052
			flexTRE+EX	-0.57	-1.29 to 0.15	0.040			
		Perimenopausal	flexTRE	0.80	-0.10 to 1.71	0.022	0.48	-0.45 to 1.41	0.183
			EX	1.56	1.08 to 2.05	**<0.001***	1.24	0.79 to 1.70	**<0.001***
			flexTRE+EX	0.32	-0.12 to 0.76	0.058			
		Postmenopausal	flexTRE	0.59	-1.03 to 2.21	0.345	1.01	-0.59 to 2.60	0.104
			EX	0.30	-0.80 to 1.40	0.476	0.72	-0.33 to 1.76	0.077
			flexTRE+EX	-0.41	-1.72 to 0.89	0.416			
QUICKI	P<0.001	Premenopausal	flexTRE	-0.01	-0.03 to 0.01	0.159	-0.03	-0.05 to -0.005	**0.002***
			EX	-0.003	-0.02 to 0.02	0.686	-0.02	-0.04 to 0.003	0.022
			flexTRE+EX	0.02	-0.01 to 0.04	0.052			
		Perimenopausal	flexTRE	-0.03	-0.07 to 0.01	0.084	0.001	-0.06 to 0.06	0.979
			EX	-0.06	-0.09 to -0.03	**<0.001***	-0.03	-0.08 to 0.02	0.126
			flexTRE+EX	-0.03	-0.09 to 0.03	0.206			
		Postmenopausal	flexTRE	-0.004	-0.06 to 0.05	0.845	-0.04	-0.09 to 0.01	0.033
			EX	0.01	-0.03 to 0.05	0.515	-0.03	-0.07 to 0.01	0.082
			flexTRE+EX	0.04	-0.02 to 0.10	0.079			

HbA1c mmol/mol	P<0.001	Premenopausal	flexTRE	-0.68	-2.53 to 1.16	0.339	-0.10	-2.31 to 2.11	0.908
			EX	-0.05	-2.33 to 2.22	0.953	0.53	-2.01 to 3.07	0.589
			flexTRE+EX	-0.58	-2.48 to 1.31	0.427			
		Perimenopausal	flexTRE	2.09	0.11 to 4.06	**0.006***	-2.28	-3.95 to -0.62	**<0.001***
			EX	0.89	-0.82 to 2.61	0.179	-3.47	-4.73 to -2.22	**<0.001***
			flexTRE+EX	4.37	2.30 to 6.44	**<0.001***			
		Postmenopausal	flexTRE	3.54	-3.90 to 10.98	0.220	1.85	-5.39 to 9.09	0.511
			EX	1.25	-2.35 to 4.85	0.370	-0.44	-3.56 to 2.68	0.717
			flexTRE+EX	1.69	-1.10 to 4.48	0.119			
Systolic BP mmHg	P<0.001	Premenopausal	flexTRE	-11.38	-21.09 to -1.68	**0.003***	-5.03	-14.90 to 4.84	0.189
			EX	-3.19	-12.92 to 6.54	0.398	3.16	-6.58 to 12.91	0.403
			flexTRE+EX	-6.35	-14.64 to 1.94	0.048			
		Perimenopausal	flexTRE	0.68	-9.62 to 10.99	0.865	12.97	-1.63 to 27.58	0.022
			EX	-17.80	-27.34 to -8.25	**<0.001***	-5.50	-20.07 to 9.07	0.331
			flexTRE+EX	-12.29	-29.25 to 4.67	0.062			
		Postmenopausal	flexTRE	5.87	-17.72 to 29.46	0.522	11.36	1.28 to 21.45	**0.004***
			EX	-3.86	-28.50 to 20.79	0.687	1.64	-10.79 to 14.07	0.734
			flexTRE+EX	-5.50	-28.53 to 17.53	0.539			
Resting heart rate rpm	P<0.001	Premenopausal	flexTRE	-2.39	-7.19 to 2.42	0.201	-1.17	-6.67 to 4.32	0.583
			EX	-2.03	-6.96 to 2.89	0.287	-0.82	-6.51 to 4.87	0.710
			flexTRE+EX	-1.21	-6.31 to 3.88	0.539			
		Perimenopausal	flexTRE	-2.10	-11.32 to 7.11	0.557	-2.27	-15.58 to 11.05	0.661
			EX	19.87	12.61 to 27.13	**<0.001***	19.71	7.68 to 31.73	**<0.001***
			flexTRE+EX	0.16	-13.74 to 14.07	0.976			
		Postmenopausal	flexTRE	-3.14	-15.72 to 9.45	0.521	0.19	-13.38 to 13.75	0.972
			EX	1.56	-4.27 to 7.39	0.491	4.88	-2.67 to 12.43	0.096
			flexTRE+EX	-3.32	-11.50 to 4.86	0.295			
Total cholesterol mmol/L	P<0.001	Premenopausal	flexTRE	-0.01	-0.55 to 0.52	0.953	0.18	-0.28 to 0.63	0.322
			EX	0.09	-0.42 to 0.59	0.658	0.27	-0.14 to 0.69	0.089
			flexTRE+EX	-0.19	-0.64 to 0.26	0.282			
		Perimenopausal	flexTRE	0.32	-0.37 to 1.02	0.233	-1.19	-2.46 to 0.08	0.016
			EX	1.38	0.77 to 1.99	**<0.001***	-0.14	-1.36 to 1.09	0.776
			flexTRE+EX	1.51	0.16 to 2.86	**0.004***			
		Postmenopausal	flexTRE	-0.38	-1.33 to 0.57	0.304	0.06	-0.89 to 1.00	0.880
			EX	-0.38	-0.88 to 0.13	0.057	0.06	-0.46 to 0.58	0.769
			flexTRE+EX	-0.44	-1.04 to 0.17	0.062			

HDL cholesterol mmol/L	P<0.001	Premenopausal	flexTRE	0.01	-0.12 to 0.14	0.823	0.02	-0.10 to 0.13	0.723
			EX	-0.01	-0.16 to 0.14	0.872	-0.004	-0.15 to 0.14	0.939
			flexTRE+EX	-0.01	-0.14 to 0.13	0.919			
		Perimenopausal	flexTRE	0.18	-0.11 to 0.48	0.108	-0.03	-0.35 to 0.29	0.811
			EX	0.47	0.23 to 0.71	**<0.001***	0.26	-0.02 to 0.53	0.015
			flexTRE+EX	0.21	-0.14 to 0.57	0.119			
		Postmenopausal	flexTRE	-0.07	-0.26 to 0.12	0.354	0.01	-0.27 to 0.30	0.915
			EX	-0.07	-0.24 to 0.10	0.302	0.01	-0.25 to 0.27	0.903
			flexTRE+EX	-0.08	-0.35 to 0.18	0.432			
LDL cholesterol mmol/L	P<0.001	Premenopausal	flexTRE	-0.09	-0.53 to 0.35	0.598	0.16	-0.29 to 0.60	0.362
			EX	-0.03	-0.35 to 0.29	0.811	0.22	-0.11 to 0.55	0.091
			flexTRE+EX	-0.25	-0.55 to 0.06	0.037			
		Perimenopausal	flexTRE	0.28	-0.52 to 1.07	0.371	-0.10	-1.13 to 0.93	0.797
			EX	0.85	0.44 to 1.26	**<0.001***	0.47	-0.29 to 1.23	0.109
			flexTRE+EX	0.38	-0.47 to 1.22	0.249			
		Postmenopausal	flexTRE	-0.60	-1.64 to 0.44	0.135	0.17	-0.85 to 1.19	0.676
			EX	-0.70	-1.22 to -0.19	**<0.001***	0.07	-0.43 to 0.56	0.736
			flexTRE+EX	-0.77	-1.36 to -0.17	**<0.001***			
Adiponectin ug/ml	P<0.001	Premenopausal	flexTRE	-0.0004	-1.54 to 1.54	0.999	-0.91	-2.80 to 0.98	0.215
			EX	0.02	-1.04 to 1.08	0.962	-0.89	-2.46 to 0.68	0.144
			flexTRE+EX	0.91	-0.78 to 2.59	0.165			
		Perimenopausal	flexTRE	-1.11	-3.15 to 0.93	0.159	0.98	-0.72 to 2.68	0.138
			EX	-1.67	-3.16 to -0.18	0.004*	0.42	-0.56 to 1.40	0.270
			flexTRE+EX	-2.09	-3.53 to -0.66	**<0.001***			
		Postmenopausal	flexTRE	-0.32	-2.73 to 2.08	0.728	-0.69	-3.99 to 2.60	0.589
			EX	-0.53	-2.14 to 1.08	0.394	-0.90	-3.64 to 1.84	0.397
			flexTRE+EX	0.37	-2.58 to 3.32	0.748			
Leptin ng/ml	P<0.001	Premenopausal	flexTRE	-3.58	-8.42 to 1.25	0.056	4.24	-0.49 to 8.97	0.021
			EX	-5.36	-9.49 to -1.23	**<0.001***	2.45	-1.73 to 6.64	0.131
			flexTRE+EX	-7.82	-11.18 to -4.46	**<0.001***			
		Perimenopausal	flexTRE	-3.29	-10.76 to 4.19	0.257	3.11	-4.49 to 10.71	0.292
			EX	1.71	-4.35 to 7.76	0.468	8.10	2.27 to 13.93	**<0.001***
			flexTRE+EX	-6.40	-13.07 to 0.28	0.014			
		Postmenopausal	flexTRE	-9.52	-24.02 to 4.99	0.091	1.06	-8.09 to 10.21	0.765
			EX	-6.77	-19.36 to 5.82	0.166	3.81	-1.94 to 9.55	0.088
			flexTRE+EX	-10.58	-23.43 to 2.26	0.034			

Resistin ng/ml	P<0.001	Premenopausal	flexTRE	-0.28	-2.21 to 1.64	0.707	0.81	-1.25 to 2.87	0.309
			EX	-0.76	-3.24 to 1.72	0.427	0.33	-2.23 to 2.89	0.740
			flexTRE+EX	-1.09	-3.24 to 1.05	0.188			
		Perimenopausal	flexTRE	4.13	-0.69 to 8.95	0.027	4.34	-0.67 to 9.35	0.026
			EX	-1.76	-3.03 to -0.50	**<0.001***	-1.55	-3.73 to 0.62	0.066
			flexTRE+EX	-0.21	-2.54 to 2.12	0.819			
		Postmenopausal	flexTRE	2.46	-0.92 to 5.84	0.061	2.59	-0.73 to 5.92	0.045
			EX	1.55	-1.04 to 4.14	0.123	1.69	-0.89 to 4.27	0.092
			flexTRE+EX	-0.13	-3.20 to 2.93	0.910			
Quality of life WHOQOL-BREF	P<0.001	Premenopausal	flexTRE	-1.53	-7.36 to 4.30	0.499	-4.85	-11.48 to 1.78	0.060
			EX	4.77	-3.48 to 13.02	0.137	1.45	-7.28 to 10.18	0.669
			flexTRE+EX	3.32	-2.31 to 8.95	0.129			
		Perimenopausal	flexTRE	6.31	1.43 to 11.20	**<0.001***	2.34	-1.31 to 5.99	0.099
			EX	20.78	16.57 to 24.98	**<0.001***	16.80	13.86 to 19.75	**<0.001***
			flexTRE+EX	3.98	-0.52 to 8.47	**0.023***			
		Postmenopausal	flexTRE	5.74	-2.94 to 14.42	0.088	4.71	-5.65 to 15.07	0.241
			EX	1.16	-4.64 to 6.96	0.607	0.13	-7.92 to 8.18	0.967
			flexTRE+EX	1.03	-7.19 to 9.24	0.747			
Eating window h/day	P<0.001	Premenopausal	flexTRE	-2.30	-3.37 to -1.23	**<0.001***	0.09	-1.03 to 1.21	0.841
			EX	1.26	-0.02 to 2.55	**0.011***	3.65	2.30 to 5.00	**<0.001***
			flexTRE+EX	-2.39	-3.72 to -1.06	**<0.001***			
		Perimenopausal	flexTRE	-1.18	-3.78 to 1.42	0.241	-0.89	-3.34 to 1.56	0.350
			EX	-1.27	-3.49 to 0.96	0.143	-0.97	-3.14 to 1.19	0.246
			flexTRE+EX	-0.29	-3.41 to 2.83	0.809			
		Postmenopausal	flexTRE	-1.76	-4.19 to 0.66	0.060	1.48	-1.42 to 4.38	0.189
			EX	-0.45	-1.88 to 0.97	0.413	2.79	0.61 to 4.97	**<0.001***
			flexTRE+EX	-3.25	-5.14 to -1.35	**<0.001***			
Carbohydrate %	P<0.001	Premenopausal	flexTRE	-0.05	-0.12 to 0.02	0.050	-0.04	-0.12 to 0.03	0.150
			EX	-0.02	-0.08 to 0.04	0.342	-0.01	-0.08 to 0.06	0.662
			flexTRE+EX	-0.01	-0.08 to 0.06	0.693			
		Perimenopausal	flexTRE	0.04	-0.09 to 0.17	0.411	-0.06	-0.25 to 0.13	0.438
			EX	0.10	0.05 to 0.14	**<0.001***	-0.002	-0.14 to 0.14	0.969
			flexTRE+EX	0.10	-0.05 to 0.24	0.083			
		Postmenopausal	flexTRE	-0.10	-0.22 to 0.02	0.034	-0.07	-0.15 to 0.02	0.046
			EX	-0.03	-0.15 to 0.09	0.522	0.01	-0.07 to 0.08	0.837
			flexTRE+EX	-0.04	-0.17 to 0.10	0.486			

Fat %	P<0.001	Premenopausal	flexTRE	0.04	-0.02 to 0.09	0.090	0.03	-0.03 to 0.09	0.150
			EX	0.01	-0.03 to 0.05	0.435	0.01	-0.04 to 0.05	0.622
			flexTRE+EX	0.004	-0.04 to 0.05	0.816			
		Perimenopausal	flexTRE	-0.03	-0.14 to 0.08	0.513	0.07	-0.09 to 0.23	0.241
			EX	-0.07	-0.11 to -0.03	**<0.001***	0.03	-0.09 to 0.15	0.543
			flexTRE+EX	-0.10	-0.22 to 0.02	0.037			
		Postmenopausal	flexTRE	0.06	-0.04 to 0.16	0.127	0.06	-0.02 to 0.14	0.044
			EX	0.02	-0.08 to 0.12	0.630	0.02	-0.06 to 0.10	0.467
			flexTRE+EX	-0.004	-0.12 to 0.11	0.928			
MVPA min/week	P=0.003	Premenopausal	flexTRE	-3.17	-40.46 to 34.12	0.827	-232.93	-353.92 to -111.94	**<0.001***
			EX	127.68	68.19 to 187.17	**<0.001***	-102.08	-232.53 to 28.37	0.044
			flexTRE+EX	229.76	106.85 to 352.67	**<0.001***			
		Perimenopausal	flexTRE	-10.20	-47.02 to 26.62	0.475	-38.01	-78.37 to 2.35	**0.015***
			EX	70.49	50.55 to 90.43	**<0.001***	42.68	14.08 to 71.29	**<0.001***
			flexTRE+EX	27.81	7.16 to 48.46	**<0.001***			
		Postmenopausal	flexTRE	19.60	-39.26 to 78.45	0.391	-192.80	-320.85 to -64.74	**<0.001***
			EX	149.89	79.63 to 220.15	**<0.001***	-62.51	-189.17 to 64.16	0.204
			flexTRE+EX	212.39	95.96 to 328.83	**<0.001***			

Note: BP, blood pressure; CI, confidence interval; CON, control group; EX, aerobic exercise group; flexTRE, flexible-time restricted eating group; flexTRE+EX, flexible time-restricted eating combined with aerobic exercise group; HbA1c, hemoglobin A1c; HDL, high-density lipoprotein; HOMA-IR, homeostatic model assessment of insulin resistance; LDL, low-density lipoprotein; MVPA, moderate-to-vigorous physical activity; QUICKI, quantitative insulin sensitivity check index; SD, standard deviation; WHOQOL-BREF, World Health Organization Quality of Life Brief Version.

The subgroup analysis was conducted to explore whether the intervention effects differed based on menopausal status (pre-menopausal, peri-menopausal, and post-menopausal) by generalized estimating equation (GEE) model. When a significant interaction effect between menopausal status-by-group-by-time was identified, we further analyzed this outcome within subgroups. An asterisk (*) in addition to a bold P-value indicates a significant pairwise difference between intervention groups over time after applying the Holm procedure for multiple comparisons.

Supplementary Table S2. Generalized Estimating Equation (GEE) Analysis of Primary, Secondary, and Additional Outcomes: Per-Protocol Population (N=98)

Table S2. Generalized Estimating Equation (GEE) Analysis of Primary, Secondary, and Additional Outcomes: Per-Protocol Population (N=98)

	Measurement	Group	Baseline			Postintervention			Time effect	Group effect	Time × group effect	Postintervention					
												Compared with CON			Compared with flexTRE+EX		
			n	Mean	SD	n	Mean	SD				Adjusted mean difference	99%CI	P value	Adjusted mean difference	99%CI	P value
**Primary outcome**																	
	Fat mass	flexTRE	24	26.69	6.68	24	25.46	6.73				-1.29	-2.33 to -0.25	**0.001***	1.56	0.38 to 2.73	**<.001***
	kg	EX	24	26.04	5.43	24	25.16	5.55	**P<0.001**	**P<0.001**	**P<0.001**	-0.84	-1.90 to 0.21	**0.039***	2.00	0.79 to 3.21	**<.001***
		flexTRE+EX	25	25.62	6.21	25	22.93	5.30				-2.85	-4.00 to -1.69	**<.001***			
		CON	25	27.50	9.23	25	27.70	9.48									
**Secondary outcomes**																	
**Other body composition parameters**	Body mass	flexTRE	24	69.15	7.69	24	67.63	7.77				-1.59	-2.50 to -0.67	**<.001***	1.78	0.64 to 2.92	**<.001***
	kg	EX	24	67.47	8.15	24	66.49	8.28	**P<0.001**	**P<0.001**	**P<0.001**	-1.28	-2.20 to -0.35	**<.001***	2.09	0.91 to 3.27	**<.001***
		flexTRE+EX	25	68.25	9.11	25	65.21	8.35				-3.37	-4.50 to -2.23	**<.001***			
		CON	25	69.46	11.15	25	69.83	11.32									
	Body mass index	flexTRE	24	27.38	3.63	24	26.75	3.65				-0.79	-1.11 to -0.47	**<.001***	0.64	0.14 to 1.13	**<.001***
	kg/m^2	EX	24	26.93	2.66	24	26.54	2.69	**P<0.001**	**P<0.001**	**P<0.001**	-0.50	-0.85 to -0.15	**<.001***	0.93	0.42 to 1.44	**<.001***
		flexTRE+EX	25	26.30	2.78	25	25.07	2.50				-1.43	-1.91 to -0.95	**<.001***			
		CON	25	27.33	3.57	25	27.48	3.62									
	Body fat percentage	flexTRE	24	38.11	5.60	24	37.15	5.69				-1.14	-2.10 to -0.19	**0.002***	1.26	0.03 to 2.48	**0.008***
	%	EX	24	38.29	3.79	24	37.48	4.04	**P<0.001**	**P<0.001**	**P<0.001**	-0.74	-1.45 to -0.04	**0.006***	1.66	0.69 to 2.63	**<.001***
		flexTRE+EX	25	37.10	3.84	25	34.80	3.61				-2.4	-3.35 to -1.45	**<.001***			
		CON	25	38.84	5.54	25	38.94	5.59									

	Fat free mass	flexTRE	24	42.46	2.85	24	42.16	2.98				-0.43	-0.99 to 0.13	0.048	0.06	-0.58 to 0.71	0.801
	kg	EX	24	41.22	3.51	24	41.33	3.56	**P=0.032**	**P=0.002**	**P=0.015**	-0.26	-0.57 to 0.06	0.034	0.24	-0.17 to 0.64	0.132
		flexTRE+EX	25	42.61	3.38	25	42.29	3.52				-0.49	-0.89 to -0.09	**0.002***			
		CON	25	41.98	3.21	25	42.12	3.14									
	Waist circumference	flexTRE	24	89.43	6.92	24	84.63	6.80				-3.25	-6.08 to -0.42	**0.003***	2.93	-0.01 to 5.87	**0.010***
	cm	EX	24	88.40	5.40	24	83.75	5.65	**P<0.001**	**P<0.001**	**P<0.001**	-3.2	-5.63 to -0.76	**<.001***	2.98	0.44 to 5.52	**0.002***
		flexTRE+EX	25	87.70	8.44	25	80.24	8.07				-6.18	-8.81 to -3.55	**<.001***			
		CON	25	90.70	9.26	25	89.04	8.07									
	Hip circumference	flexTRE	24	106.56	7.48	24	101.92	6.01				-3.27	-5.57 to -0.96	**<.001***	1.95	-0.44 to 4.35	0.036
	cm	EX	24	104.79	4.84	24	99.83	4.52	**P<0.001**	**P<0.001**	**P<0.001**	-3.65	-5.82 to -1.48	**<.001***	1.57	-0.75 to 3.88	0.081
		flexTRE+EX	25	104.40	6.27	25	98.08	5.35				-5.22	-7.75 to -2.69	**<.001***			
		CON	25	106.28	9.46	25	105.12	8.99									
**Glycemic control**	Fasting glucose	flexTRE	24	5.46	0.80	24	5.43	0.72				0.05	-0.18 to 0.28	0.576	0.09	-0.18 to 0.36	0.395
	mmol/L	EX	24	5.39	0.42	24	5.45	0.44	P=0.469	P=0.351	P=0.477	0.14	-0.12 to 0.40	0.169	0.18	-0.11 to 0.46	0.111
		flexTRE+EX	25	5.36	0.56	25	5.21	0.48				-0.04	-0.31 to 0.23	0.707			
		CON	25	5.22	0.48	25	5.18	0.50									
	Insulin	flexTRE	24	8.67	5.48	24	9.77	5.12				1.43	-0.82 to 3.69	0.102	2.99	0.91 to 5.06	**<.001***
	mU/L	EX	24	8.71	5.03	24	9.14	3.56				1.25	-1.30 to 3.79	0.207	2.80	0.38 to 5.22	**0.003***
		flexTRE+EX	25	8.44	6.23	25	6.52	3.64	P=0.084	**P=0.005**	**P=0.018**	-1.55	-3.69 to 0.58	0.061			
		CON	25	8.64	5.16	25	8.54	5.22									
	HOMA-IR	flexTRE	24	2.17	1.59	24	2.46	1.59				0.35	-0.24 to 0.93	0.126	0.73	0.19 to 1.26	**<.001***
		EX	24	2.07	1.16	24	2.21	0.90				0.36	-0.27 to 1.00	0.141	0.74	0.14 to 1.34	**0.001***
		flexTRE+EX	25	2.11	1.86	25	1.52	0.89	p=0.118	**P=0.006**	**p=0.023**	-0.38	-0.92 to 0.17	0.074			
		CON	25	2.06	1.34	25	2.03	1.38									

	QUICKI	flexTRE	24	0.36	0.04	24	0.35	0.03				-0.01	-0.03 to 0.01	0.155	-0.03	-0.05 to -0.006	**<.001***
		EX	24	0.35	0.03	24	0.34	0.02				-0.01	-0.02 to 0.01	0.360	-0.02	-0.04 to -0.003	**0.003***
		flexTRE+EX	25	0.36	0.04	25	0.38	0.05	P=0.949	**P=0.007**	**P=0.010**	0.02	-0.01 to 0.04	0.061			
		CON	25	0.36	0.05	25	0.36	0.04									
	HbA1c	flexTRE	24	36.42	4.75	24	36.08	5.56				0.68	-1.23 to 2.60	0.359	0.13	-1.87 to 2.12	0.872
	mmol/mol	EX	24	37.17	3.42	24	36.29	3.59				0.30	-1.71 to 2.32	0.698	-0.25	-2.31 to 1.81	0.753
		TRE+EX	25	36.24	4.71	25	35.72	3.85	P=0.019	P=0.573	P=0.847	0.56	-0.99 to 2.10	0.353			
		CON	25	35.24	3.63	25	34.32	3.64									
**Cardiometabolic parameters**	Systolic BP	flexTRE	24	108.06	13.97	24	101.08	18.60				-5.72	-14.69 to 3.25	0.101	0.93	-6.78 to 8.65	0.755
	mmHg	EX	24	111.35	15.79	24	104.33	16.75	P<0.001	P=0.229	P=0.204	-5.36	-15.24 to 4.51	0.162	1.29	-6.67 to 9.24	0.677
		flexTRE+EX	25	110.66	13.09	25	102.28	13.56				-6.65	-14.85 to 1.55	0.037			
		CON	25	107.68	15.12	25	106.48	12.25									
	Diastolic BP	flexTRE	24	76.81	9.06	24	76.17	10.67				-0.80	-8.27 to 6.67	0.782	2.01	-5.23 to 9.24	0.475
	mmHg	EX	24	75.83	10.59	24	75.42	13.60				-1.41	-9.09 to 6.28	0.637	1.40	-5.77 to 8.57	0.615
		flexTRE+EX	25	76.86	7.32	25	74.48	11.37	P=0.427	P=0.712	P=0.797	-2.81	-9.90 to 4.28	0.307			
		CON	25	77.76	11.41	25	77.88	13.22									
	Resting heart rate	flexTRE	24	72.17	9.16	24	69.50	6.14				-2.43	-6.51 to 1.64	0.124	-0.69	-5.39 to 4.01	0.706
	rpm	EX	24	70.48	9.04	24	70.88	8.62				0.73	-3.49 to 4.96	0.655	2.48	-2.30 to 7.26	0.181
		flexTRE+EX	25	72.58	8.80	25	70.36	7.98	P=0.069	P=0.206	P=0.350	-1.75	-5.88 to 2.39	0.276			
		CON	25	72.12	7.93	25	71.76	8.78									
	Total cholesterol	flexTRE	24	5.77	0.94	24	5.66	0.77				-0.05	-0.47 to 0.37	0.760	0.02	-0.40 to 0.44	0.899
	mmol/L	EX	24	5.90	0.75	24	5.91	0.88	P=0.337	P=0.825	P=0.887	0.04	-0.34 to 0.42	0.767	0.11	-0.26 to 0.49	0.431
		flexTRE+EX	25	5.50	0.83	25	5.42	0.89				-0.07	-0.48 to 0.34	0.664			
		CON	25	5.58	0.96	25	5.55	1.13									

	HDL cholesterol	flexTRE	24	1.43	0.35	24	1.41	0.29				0.02	-0.08 to 0.13	0.580	0.02	-0.09 to 0.13	0.641
	mmol/L	EX	24	1.47	0.33	24	1.44	0.35				0.01	-0.11 to 0.13	0.818	0.01	-0.12 to 0.13	0.882
		flexTRE+EX	25	1.57	0.36	25	1.52	0.29	P=0.017	P=0.998	P=0.803	0.004	-0.12 to 0.12	0.940			
		CON	25	1.57	0.32	25	1.51	0.31									
	LDL cholesterol	flexTRE	24	3.63	0.74	24	3.55	0.80				-0.14	-0.53 to 0.24	0.335	0.16	-0.22 to 0.55	0.278
	mmol/L	EX	24	3.72	0.64	24	3.62	0.71				-0.18	-0.49 to 0.13	0.130	0.12	-0.17 to 0.42	0.277
		flexTRE+EX	25	3.24	0.69	25	3.04	0.67	P=0.158	P=0.077	P=0.089	-0.31	-0.61 to -0.003	0.009			
		CON	25	3.35	0.68	25	3.44	0.87									
	Triglycerides	flexTRE	24	1.03	0.39	24	1.13	0.37				0.15	-0.11 to 0.40	0.135	0.20	-0.04 to 0.45	0.032
	mmol/L	EX	24	1.36	0.52	24	1.28	0.52				0.04	-0.23 to 0.230	0.719	0.10	-0.17 to 0.36	0.361
		flexTRE+EX	25	1.30	1.00	25	1.21	0.70	P=0.404	P=0.059	P=0.522	-0.06	-0.29 to 0.18	0.522			
		CON	25	1.22	0.63	25	1.16	0.49									
Adipokines	Adiponectin	flexTRE	24	4.97	3.03	24	5.21	2.36				-0.27	-1.47 to 0.93	0.564	-0.76	-2.34 to 0.83	0.219
	ug/ml	EX	24	5.50	2.42	24	5.46	1.82				-0.35	-1.21 to 0.51	0.299	-0.83	-2.20 to 0.53	0.116
		flexTRE+EX	25	5.89	4.27	25	6.71	3.82	P<0.001	P=0.391	P=0.134	0.49	-0.95 to 1.92	0.381			
		CON	25	5.93	2.24	25	6.36	2.83									
	Leptin	flexTRE	24	19.24	12.85	24	16.49	13.21				-4.73	-9.26 to -0.19	**0.007***	3.49	-0.19 to 7.18	**0.015***
	ng/ml	EX	24	19.86	10.29	24	14.43	5.64				-4.89	-9.23 to -0.54	**0.004***	3.33	0.12 to 6.55	**0.008***
		flexTRE+EX	25	16.21	5.26	25	9.99	5.10	**P<0.001**	**P<0.001**	**P<0.001**	-8.22	-11.88 to -4.56	**<.001***			
		CON	25	21.02	13.00	25	22.45	14.33									
	Resistin	flexTRE	24	4.63	2.91	24	5.82	4.14				1.03	-0.77 to 2.83	0.142	1.80	-0.09 to 3.69	0.014
	ng/ml	EX	24	3.82	2.18	24	3.60	2.03				0.11	-1.69 to 1.90	0.877	0.88	-0.97 to 2.72	0.220
		flexTRE+EX	25	5.84	5.39	25	4.69	4.00	P=0.134	P=0.433	**P=0.026**	-0.77	-2.48 to 0.94	0.245			
		CON	25	6.62	6.23	25	6.94	6.30									

**Questionnaires**	Sleep quality	flexTRE	24	6.54	2.95	23	6.48	3.31				0.05	-2.03 to 2.13	0.949	-0.90	-3.32 to 1.51	0.335
	PSQI	EX	23	7.26	2.49	24	6.33	2.84	P=0.612	P=0.117	P=0.243	-0.97	-2.92 to 0.98	0.200	-1.92	-4.18 to 0.33	0.028
		flexTRE+EX	25	6.68	3.52	24	7.58	3.67				0.96	-1.33 to 3.25	0.283			
		CON	25	7.44	3.11	25	7.12	3.59									
	Quality of life	flexTRE	24	90.04	13.17	24	90.46	13.09				1.01	-3.36 to 5.39	0.551	-1.98	-7.08 to 3.13	0.318
	WHOQOL-BREF	EX	24	87.00	9.91	24	91.13	10.99	P=0.018	P=0.123	P=0.078	4.29	-0.68 to 9.27	0.026	1.30	-4.23 to 6.84	0.544
		flexTRE+EX	25	89.56	11.55	24	92.08	12.60				2.99	-1.34 to 7.32	0.075			
		CON	24	85.63	12.05	25	85.52	12.61									
	Mood profile	flexTRE	23	126.13	17.96	24	127.83	18.24				5.65	-5.16 to 16.46	0.178	-2.02	-13.64 to 9.61	0.655
	POMS	EX	21	120.57	14.76	24	121.38	13.87	P=0.563	P=0.181	P=0.355	0.96	-8.77 to 10.70	0.798	-6.70	-17.61 to 4.21	0.114
		flexTRE+EX	25	123.32	12.41	25	128.32	21.10				7.67	-4.03 to 19.36	0.091			
		CON	24	125.29	16.94	25	123.44	13.69									
**Monitor parameters**	Energy intake	flexTRE	24	7185.32	608.14	24	6361.06	742.20				-797.90	-1289.74 to -306.06	**<.001***	200.00	-379.27 to 779.28	0.374
	kJ/day	EX	24	7135.81	471.80	24	6863.54	587.50	**p<.001**	**p<.001**	**p<.001**	-278.75	-728.40 to 170.90	0.110	719.16	169.75 to 1268.56	**<.001***
		flexTRE+EX	25	7131.29	360.79	25	6154.50	754.59				-997.91	-1504.61 to -491.21	**<.001***			
		CON	25	6922.22	760.66	25	7024.74	626.74									
	Eating window	flexTRE	24	10.33	1.11	24	7.88	1.47				-2.00	-2.94 to -1.06	**<.001***	0.43	-0.67 to 1.53	0.317
	h/day	EX	24	10.46	1.44	24	10.19	1.95	**p<.001**	**p<.001**	**p<.001**	0.35	-0.70 to 1.40	0.392	2.78	1.56 to 4.00	**<.001***
		flexTRE+EX	25	10.70	1.72	25	7.64	1.64				-2.43	-3.53 to -1.33	**<.001***			
		CON	25	10.94	1.53	25	10.32	1.41									
	Carbohydrate %	flexTRE	24	0.45	0.06	24	0.42	0.07				-0.05	-0.11 to 0.01	0.042	-0.04	-0.10 to 0.02	0.085
		EX	24	0.47	0.09	24	0.47	0.07	p=0.530	p=0.149	p=0.112	-0.01	-0.06 to 0.04	0.571	-0.005	-0.05 to 0.04	0.786
		flexTRE+EX	25	0.44	0.07	25	0.46	0.08				-0.006	-0.06 to 0.05	0.799			
		CON	25	0.42	0.07	25	0.45	0.07									

Protein %	flexTRE	24	0.18	0.04	24	0.19	0.04				0.02	-0.01 to 0.05	0.091	0.002	-0.03 to 0.03	0.868
	EX	24	0.17	0.04	24	0.17	0.04	p=0.954	p=0.221	p=0.125	0.002	-0.02 to 0.02	0.844	-0.015	-0.04 to 0.01	0.115
	flexTRE+EX	25	0.17	0.03	25	0.18	0.04				0.02	-0.01 to 0.04	0.074			
	CON	25	0.19	0.04	25	0.18	0.04									
Fat %	flexTRE	24	0.37	0.05	24	0.39	0.06				0.03	-0.02 to 0.08	0.114	0.04	-0.01 to 0.09	0.055
	EX	24	0.36	0.07	24	0.37	0.07	p=0.430	p=0.356	p=0.089	0.01	-0.03 to 0.05	0.550	0.02	-0.02 to 0.06	0.271
	flexTRE+EX	25	0.39	0.06	25	0.36	0.07				-0.01	-0.05 to 0.04	0.623			
	CON	25	0.39	0.05	25	0.37	0.06									
MVPA	flexTRE	24	56.34	55.21	23	63.16	45.05				1.44	-24.69 to 27.57	0.887	-201.56	-291.90 to -111.22	**<.001***
min/week	EX	24	79.79	64.17	23	209.42	88.62	**p<.001**	**p<.001**	**p<.001**	130.16	86.48 to 173.84	**<.001***	-72.84	-166.14 to 20.46	0.044
	flexTRE+EX	25	61.73	62.05	21	211.01	112.43				203.00	113.11 to 292.89	**<.001***			
	CON	25	58.72	73.86	24	62.44	49.18									

Note: BP: blood pressure; CI: confidence interval; CON: control group; EX: aerobic exercise; flexTRE: flexible time-restricted eating; flexTRE+EX: flexible time-restricted eating combined with aerobic exercise group; HbA1c: Hemoglobin A1c; HDL: high-density lipoprotein; HOMA-IR: Homeostatic Model Assessment of Insulin Resistance; LDL: low-density lipoprotein; MVPA: moderate-to-vigorous physical activity; POMS: Profile of Mood States; PSQI: Pittsburgh Sleep Quality Index; QUICKI: Quantitative insulin sensitivity check index; SD: standard deviation; WHOQOL-BREF: World Health Organization Quality of Life Brief Version.

The secondary per-protocol analyses included only those participants who completed all baseline and post-intervention assessments. Ninety-eight participants (flexTRE, n=24; EX, n=24; flexTRE+EX, n=25; CON, n=25) were included in the PP analyses. Intervention effects on these outcomes were examined by generalized estimation equations (GEE). The model assessed the main effects of group and time, as well as the group-by-time interaction, while adjusting for covariates including baseline outcome values, age, menopausal status, and family history of cardiovascular disease. A significant group-by-time interaction indicated a significant difference for a given outcome between interventions over time. Pairwise treatment comparisons were performed by linear contrasts using the Holm procedure to adjust for multiple comparisons, and raw P values are presented. Bold P-values indicate a significant main effect of time, group, or a group-by-time interaction ($P < 0.05$). An asterisk (*) in addition to a bold P-value indicates a significant pairwise difference between intervention groups over time after applying the Holm procedure for multiple comparisons.

Supplementary Note 1

Study Protocol: Effects of Time-restricted Eating Combined with Aerobic Exercise on Body Composition and Metabolic Health in Middle-aged Overweight/Obese Females

Background

Obesity is a multifactorial disease currently comprising a global public health concern, increasing the risk of non-communicable diseases, such as diabetes and cardiovascular disease [1]. According to the World Health Organization, it is estimated that more than 1 billion people worldwide are obese, and this number is still increasing [2]. Recently, a new world obesity atlas report from the World Obesity Federation, predicts that 51% of the global population will be living with overweight or obesity by 2035 based on current trends. The majority of the global population (51%, or over 4 billion people) will be living with either overweight or obesity by 2035 if the current trends prevail. According to this report, females show a higher prevalence of obesity than men and keep increasing [3]. Another study finds that midlife women gain weight at an average of approximately 1.5 pounds per year, which increases their risk for transitioning from normal or overweight to obese BMI. Women between 40 and 60 years old are the focus of new national guidelines aimed at preventing unhealthy weight gain that can lead to serious illness [4]. A practical and sound life modification for the treatment of overweight and obesity is still necessary.

Recently, intermittent fasting (IF) has gained attention as a simple weight loss method. Intermittent fasting refers to eating windows separated by defined periods of fasting (> 12 hours and up to 48 hours, or more) [5]. Time-restricted eating (TRE) is a specific IF protocol involving consistent fasting and eating periods within a 24-hour cycle [6]. TRE involves confining the eating window to a specified number of hours per day (usually 3-12 hours) and fasting with zero-calorie beverages for the remaining hours of the day [7]. TRE has been shown to be an effective and realistic strategy for losing body weight and improving general health and sustaining optimal nutritional value utilization [8,9]. While TRE places no restriction on total energy intake or the macronutrient composition of food, individuals often spontaneously reduce their energy intake by 20%-30%, inducing a mild (1%-4%) body weight loss over intervention periods lasting from 1 week to 12 weeks without the need to count calories [7].

TRE extends the interval between the final meal of the day and the first meal of the following day [10]. When the eating window for energy intake is shortened to less than 8-10 hours per day from 12-14 hours, obesity and other metabolic health parameters are improved in both rodent models [11] and humans [8,12-17], and TRE has been proved to be a feasible and practical strategy to improve cardiometabolic health and improve overall body composition in overweight or obesity male individuals [16] and females [5]. In addition to the overweight and obese population, TRE also shows the benefits of metabolic health in males with prediabetes [17]; individuals with metabolic syndrome [18]; with type 2 diabetes [19], as well as healthy individuals without obesity [20].

Current research in TRE has been separated into different types, the eating windows vary from 4h, 6h, 8h, and 10h TRE. The comparison of TRE between different eating windows is still lacking evidence, each eating window has its benefit on body composition and metabolic health. Most of the TRE has been investigated confined to the eating window of 8 hours, and 16/8 TRE is a reasonable and popular type accepted by the general population. One recent study also found that 12 weeks of 8h TRE can induce a weight loss at 4.1% and decrease the

diastolic blood pressure in the middle-aged female [15].

Aerobic exercise refers to any activity that uses large muscle groups, that can be maintained continuously and is rhythmic in nature [21]. Aerobic exercise stimulates the heart, lungs, and all working groups of muscles and produces beneficial changes in body and mind. Many physiological changes are determined by regular aerobic exercises. Aerobic exercise has also been used as a non-pharmacological intervention to prevent or treat disorders related to overweight and obesity [22]. Aerobic exercise has shown strong evidence for reducing body weight and fat mass [23]; improving glucose metabolism and lipid profile [24]; improving inflammatory markers in middle-aged adults [25] and improving quality of life, mood, and mental health in individuals with obesity [26].

Diet modification and exercise training are primary lifestyle strategies for obesity and overweight [27]. TRE and aerobic exercise both improve cardiometabolic health in at-risk individuals, but whether these two interventions combined induce superior improvements in body composition and other metabolic health than each individual intervention is not known. In a rodent study, the mice fed a high-fat diet, time-restricted feeding combined with aerobic exercise training attenuated fat mass gain and adverse metabolic changes in lipid metabolism, insulin signaling, and glycemic control [28]. However, there exists a significant difference between robust experience and human study, whether results from animal experiments can be applied to the human body should be further assessed.

Previous human investigations have determined the effect of combined TRE and endurance exercise training on body composition and exercise performance in trained individuals and athletes [29-31], but little is known about the metabolic benefits of this combined therapy in overweight/obese middle-aged females. Recently, a study has combined TRE and high-intensity interval training in reproductive-aged women, this study found that the combination therapy decreases HbA1c and reduces more visceral fat than TRE and HIIT alone which elucidates that TRE and HIIT are feasible strategies to improve metabolic health in this population [32]. However, this study only has 7 weeks for the intervention, which may have been too short to induce substantial changes in markers of glycemic control and other metabolic health outcomes.

Accordingly, in this four-armed randomized controlled trial, we aim to examine the isolated and combined effects of 12 weeks of TRE (≤ 8-h daily eating window, with ad libitum energy intake) and Aerobic exercise (three exercise sessions per week), compared with a non-intervention control group, on body composition and other metabolic health outcomes in middle-aged females with overweight/obesity in a free-living setting. It is hypothesized that aerobic exercise combined with TRE will be more effective than individual groups on body composition and other metabolic markers in middle-aged overweight/obese females.

Method

Participants:

Participants will be recruited if they meet the following inclusion criteria: 1) Chinese females aged 40-60 years old; 2) Obesity/overweight: body mass index ≥ 23kg/m^2; 3) physically inactive; 4) Weight stable for 3 months prior to the beginning of the study (gain or loss < 4kg); 5) Baseline eating period is ≥ 12 h per day. This sample size was justified by a prior power analysis using a target effect size of 0.25, alpha of 0.05, and power of 0.95 with fat mass as

an outcome measure based on previously established results [33], subjects should be more than 76, considering of the 15% drop rate, at least 89 participants will be recruited.

The exclusion criteria of participants include 1) Known cardiovascular disease; 2) Type 1 or 2 diabetes mellitus; 3) Currently taking medications that could affect study outcomes. For example, antihypertension medication, glucose-lowering or lipid-lowering medication; 4) Known musculoskeletal disease or injury that will affect the exercise; 5) Night shift workers; 6) Breastfeeding, pregnant, or trying to become pregnant; 7) Smokers; 8) Not currently on a special or prescribed diet for other reasons.

Study design:

This study will be a single-center, parallel-group, assessor-blinded, four-armed randomized controlled trial investigating the isolated and combined effects of 12 weeks of TRE and Aerobic exercise, compared with a non-intervention control group in a free-living setting on body composition and metabolic health in middle-aged overweight/obese females. Assessments will be performed before and immediately after the intervention.

Participant recruitment and randomization:

Participants will be recruited from the general community through various means, including advertisement via posters on campus and social media. An online questionnaire will be developed for preliminary screening, covering physical activity levels, health status, and obesity status. Following the completion of the preliminary screening through an online questionnaire, potential participants will undergo a screening visit at the laboratory. During this visit, participants will be required to complete the Physical Activity Readiness Questionnaire, the International Physical Activity Questionnaire, and a health history questionnaire. Anthropometric assessments will be conducted by a trained researcher to measure body mass and height for BMI calculation. Participants will be instructed to maintain their regular diet and physical activity routines for one week. Throughout this period, participants will be asked to record their daily eating window and energy intake for 24 hours over seven consecutive days. Those meeting the inclusion criteria will be invited to take part in the study. All participants will be provided written informed consent prior to enrollment. After the baseline assessment, an independent researcher will utilize an online random number generator to allocate participants to the four study groups in a 1:1:1:1 ratio with a block size of 8. Randomization lists will be generated and sealed in envelopes, which will be opened by a research assistant upon receipt of written informed consent from the participants. The outcome assessors will be blinded to the group allocation.

Intervention protocol:

Control group (waiting list):

Participants in the control group will be required to maintain their normal levels of physical activity and food habits. The control group will be called once a week to ensure that they are keeping up with their registration and adherence. Participants in this group will be provided another treatment after the 12-week intervention if they want, they can choose from the other three intervention groups and receive a 12-week supervised research intervention based on their preference.

Time-restricted eating group:

Participants in the TRE group will be advised to follow an ad libitum eating pattern within an 8-hour eating window, employing the 16/8 intermittent fasting regimen. No restrictions on

food types or quantities will be imposed during the 8-hour eating period, and participants will not be required to monitor energy intake. Throughout fasting periods, participants will be encouraged to stay hydrated with water and allowed energy-free beverages like black tea and coffee. Participants will have the flexibility to select their preferred eating window, ranging from 8 am to 12 pm for commencement and ending at 4 pm to 8 pm. To enhance adherence, participants will be expected to log their daily eating start and stop times online, with researchers monitoring logs daily and sending reminders to non-compliant participants.

Aerobic exercise group (EX):
The aerobic exercise group will exercise 3 times for non-consecutive days for 12 weeks. As most inactive participants may have a relatively low fitness level, they will apply a progressive approach of the designated exercise time duration in this intervention.

Week1 – Week2: 30 min of easy jogging or brisk walking @ 64-76% HRmax
Week3 – Week12: 40 min of easy jogging or brisk walking @ 64-76% HRmax

Each training session will also include a 5-minute warm-up and a 5-minute walk cool down. The total exercise time is designated to accumulate about 150 min of moderate-intensity exercise per week, in accordance with PA guidelines. Running, jogging, or brisk walking on a treadmill or outdoors are allowed, as long as achieve the prescribed heart rate. Participants will be provided with heart rate monitors (Polar, Finland) and the training session data will be uploaded to the Polar Flow website via the Polar Flow app on the participants' phones. Data will be collected every week to check if the participants meet the aerobic exercise intervention and provide timely feedback on how to maintain adherence.

Combination of TRE and Aerobic exercise group (TRE+EX):
The participants in this group will undergo diet and exercise interventions together, following the TRE regimen and Aerobic exercise plan mentioned above. The participants in this group need to consume all calories in a self-selected 8-hour eating window. Considering the practical situation, it will be hard to arrange a specific fixed time for the participants to exercise. Subjects can arrange their own time to do aerobic exercise, but the exercise time should be between 8 am to 10 pm to mitigate the risk of hypoglycemia.

Adherence:
Adherence to TRE was computed as the percentage of days with an eating window of $\leqslant$ 8 hours over the 12-week (84 days) intervention period. For the aerobic exercise group, adherence was determined as the percentage of completed exercise sessions out of the total of 36 sessions (12 weeks * 3 sessions/week).

Outcome Measurements:
Primary outcome measure
Fat mass: Fat mass will be evaluated using a bioelectrical impedance analyzer (MC-780 MA, Tanita, Japan). The assessment will follow a standardized protocol and conduct at the same time in the morning, with participants wearing light, form-fitting attire and barefoot.

Secondary outcome measure
Other Body composition outcomes: The height and body composition including body mass, body mass index, body fat percentage, and fat-free mass of each participant will be measured using a stadiometer (Seca, Leicester, UK) and a bioelectrical impedance analyzer (MC-780

MA, Tanita, Japan), respectively. Waist circumference and hip circumference will be using a tension tape to the nearest 0.1 cm.

Metabolic health markers measurements:
Fasting blood samples will be collected at baseline and post-intervention. The tubes will be centrifuged (Thermo Scientific™, Waltham, USA) for 12 min at 3500 rpm at 4 ºC. The separate plasma and serum will be collected in Eppendorf tubes and stored at -80℃ in an ultra-low temperature freezer (Sanyo™, Osaka, Japan) for further analysis.

Lipid profile, including total cholesterol, triglyceride, high-density lipoprotein cholesterol (HDL), and low-density lipoprotein cholesterol (LDL) will be measured using a Cobas c 111 analyzer (Roche Diagnostics, Rotkreuz, Switzerland). Adipokine concentrations will be measured using ELISA kits (R&D Systems, Minneapolis, US).

Glucoregulatory factors include fasting glucose, HbA1c, fasting insulin, HOMA-IR and QUICKI. Plasma glucose concentrations and HbA1c levels will be promptly measured post-blood sample collection using portable analyzers (Contour Plus Glucometer, Bayer Healthcare, Germany) and the Cobas B 101 HbA1c testing system (Roche Diagnostics, Rotkreuz, Switzerland), respectively. Insulin will be measured using commercial ELISA kits (Mercodia, Uppsala, Sweden). HOMA-IR and QUICKI used the formula HOMA-IR= [fasting insulin (μg/ml)]*[fasting glucose (mmol/l)]/22.5 and QUICKI=1/[log(fasting insulin (μU/ml) + log(fasting glucose (mg/dl)].

Blood pressure and heart rate will be measured using a clinical automatic blood pressure monitor (M7 Intelli IT, Omron, Japan) after a 10-min rest. The cuff will be placed around the participants' brachial artery (left arm). Two readings (with 1-min intervals) of systolic blood pressure (SBP), diastolic blood pressure (DBP), and heart rate will be averaged.

The Pittsburgh sleep quality index, WHOQOL-BREF and modified profile of mood states will be used to assess Subjective sleep, quality of life, and mood profile. The energy intake will be assessed using a validated seven-day food record during the week before and the last week of intervention. Each time, participants will be asked to record their daily energy intake with ingredients and quantities, including meals, snacks, and drinks, and send pictures of their meals to the investigators. Completed dietary records will be analyzed using the online nutrition database of the Centre for Food Safety, Hong Kong (http://www.cfs.gov.hk/). The total energy intake and macronutrient distribution will be calculated.

Statistical Analysis
All the data will be presented as mean ± standard deviation (SD) and number or percentage for continuous and categorical variables, respectively. To examine the effects of the intervention (TRE+EX vs. CON, TRE+EX vs. TRE, TRE+EX vs. EX, TRE vs. CON, and EX vs. CON) on outcomes (primary outcome and secondary outcomes), generalized estimating equations, adjusting for covariates (participants' age, family disease history, menstrual cycle status, and baseline outcome value). A significant group effect indicates a significant difference among groups in the change of particular outcomes after intervention. Statistical analyses will be performed using SPSS, version 28.0 (IBM Corp., Armonk, NY, USA), with significance set at $p<0.05$.

References

1. Chooi YC, Ding C, Magkos F. The epidemiology of obesity. *Metabolism-Clinical and Experimental*. Mar 2019;92:6-10. doi:10.1016/j.metabol.2018.09.005

2. World Health Organization. Obesity and overweight. 2022; https://www.who.int/news-room/fact-sheets/detail/obesity-and-overweight

3. World obesity federation. Economic impact of overweight and obesity to surpass $4 trillion by 2035. Mar 2023; https://www.worldobesity.org/news/economic-impact-of-overweight-and-obesity-to-surpass-4-trillion-by-2035

4. Chelmow D, Gregory KD, Witkop C, et al. Preventing Obesity in Midlife Women: A Recommendation From the Women's Preventive Services Initiative. *Annals of Internal Medicine*. Sep 2022;175(9):1305-+. doi:10.7326/m22-0252

5. Lowe DA, Wu N, Rohdin-Bibby L, et al. Effects of Time-Restricted Eating on Weight Loss and Other Metabolic Parameters in Women and Men With Overweight and Obesity The TREAT Randomized Clinical Trial. *Jama Internal Medicine*. Nov 2020;180(11):1491-1499. doi:10.1001/jamainternmed.2020.4153

6. Parr EB, Devlin BL, Hawley JA. Perspective: Time-Restricted Eating-Integrating the What with the When. *Advances in Nutrition*. Jun 2022;13(3):699-711. doi:10.1093/advances/nmac015

7. Varady KA, Cienfuegos S, Ezpeleta M, Gabel K. Clinical application of intermittent fasting for weight loss: progress and future directions. *Nature Reviews Endocrinology*. May 2022;18(5):309-321. doi:10.1038/s41574-022-00638-x

8. Kang J, Ratamess NA, Faigenbaum AD, et al. Effect of Time-Restricted Feeding on Anthropometric, Metabolic, and Fitness Parameters: A Systematic Review. *Journal of the American Nutrition Association*. Nov 2022;41(8):810-825. doi:10.1080/07315724.2021.1958719

9. Parr EB, Heilbronn LK, Hawley JA. A Time to Eat and a Time to Exercise. *Exercise and Sport Sciences Reviews*. Jan 2020;48(1):4-10. doi:10.1249/jes.0000000000000207

10. Moholdt T, Silva CP, Lydersen S, Hawley JA. Isolated and combined effects of high-intensity interval training and time-restricted eating on glycaemic control in reproductive-aged women with overweight or obesity: study protocol for a four-armed randomised controlled trial. *Bmj Open*. Feb 2021;11(2)e040020. doi:10.1136/bmjopen-2020-040020

11. Hatori M, Vollmers C, Zarrinpar A, et al. Time-Restricted Feeding without Reducing Caloric Intake Prevents Metabolic Diseases in Mice Fed a High-Fat Diet. *Cell Metabolism*. Jun 2012;15(6):848-860. doi:10.1016/j.cmet.2012.04.019

12. Gabel K, Hoddy KK, Haggerty N, Varady KA. Effect of 8-Hour Time Restricted Feeding on Body Weight in Obese Subjects. *Faseb Journal*. Apr 2017;31

13. Gill S, Panda S. A Smartphone App Reveals Erratic Diurnal Eating Patterns in Humans that Can Be Modulated for Health Benefits. *Cell Metabolism*. Nov 2015;22(5):789-798. doi:10.1016/j.cmet.2015.09.005
14. Jamshed H, Beyl RA, Della Manna DL, Yang ES, Ravussin E, Peterson CM. Early Time-Restricted Feeding Improves 24-Hour Glucose Levels and Affects Markers of the Circadian Clock, Aging, and Autophagy in Humans. *Nutrients*. Jun 2019;11(6)1234. doi:10.3390/nu11061234
15. Lin YJ, Wang YT, Chan LC, Chu NF. Effect of time-restricted feeding on body composition and cardiometabolic risk in middle-aged women in Taiwan. *Nutrition*. Jan 2022;93111504. doi:10.1016/j.nut.2021.111504
16. Parr EB, Devlin BL, Radford BE, Hawley JA. A Delayed Morning and Earlier Evening Time-Restricted Feeding Protocol for Improving Glycemic Control and Dietary Adherence in Men with Overweight/Obesity: A Randomized Controlled Trial. *Nutrients*. Feb 2020;12(2)505. doi:10.3390/nu12020505
17. Sutton EF, Beyl R, Early KS, Cefalu WT, Ravussin E, Peterson CM. Early Time-Restricted Feeding Improves Insulin Sensitivity, Blood Pressure, and Oxidative Stress Even without Weight Loss in Men with Prediabetes. *Cell Metabolism*. Jun 2018;27(6):1212-+. doi:10.1016/j.cmet.2018.04.010
18. Wilkinson MJ, Manoogian ENC, Zadourian A, et al. Ten-Hour Time-Restricted Eating Reduces Weight, Blood Pressure, and Atherogenic Lipids in Patients with Metabolic Syndrome. *Cell Metabolism*. Jan 2020;31(1):92-+. doi:10.1016/j.cmet.2019.11.004
19. Parr EB, Devlin BL, Lim KHC, et al. Time-Restricted Eating as a Nutrition Strategy for Individuals with Type 2 Diabetes: A Feasibility Study. *Nutrients*. Nov 2020;12(11)3228. doi:10.3390/nu12113228
20. Xie ZB, Sun YN, Ye YQ, et al. Randomized controlled trial for time-restricted eating in healthy volunteers without obesity. *Nature Communications*. Feb 2022;13(1)1003. doi:10.1038/s41467-022-28662-5
21. Patel H, Alkhawam H, Madanieh R, Shah N, Kosmas CE, Vittorio TJ. Aerobic vs anaerobic exercise training effects on the cardiovascular system. *World Journal of Cardiology*. Feb 2017;9(2):134-138. doi:10.4330/wjc.v9.i2.134
22. Shahana A, Nair US, Hasrani SS. Effect of aerobic exercise programme on health related physical fitness components of middle aged women. *British Journal of Sports Medicine*. 2010;44(Suppl_1):i19-i19.
23. Garrow JS, Summerbell CD. METAANALYSIS - EFFECT OF EXERCISE, WITH OR WITHOUT DIETING, ON THE BODY-COMPOSITION OF OVERWEIGHT SUBJECTS. *European Journal of Clinical Nutrition*. Jan 1995;49(1):1-10.
24. Dengel DR, Hagberg JM, Pratley RE, Rogus EM, Goldberg AP. Improvements in blood pressure, glucose metabolism, and lipoprotein lipids after aerobic exercise plus weight loss in

obese, hypertensive middle-aged men. *Metabolism-Clinical and Experimental*. Sep 1998;47(9):1075-1082. doi:10.1016/s0026-0495(98)90281-5
25. Zheng GH, Qiu PT, Xia R, et al. Effect of Aerobic Exercise on Inflammatory Markers in Healthy Middle-Aged and Older Adults: A Systematic Review and Meta-Analysis of Randomized Controlled Trials. *Frontiers in Aging Neuroscience*. Apr 2019;1198. doi:10.3389/fnagi.2019.00098
26. Carraca EV, Encantado J, Battista F, et al. Effect of exercise training on psychological outcomes in adults with overweight or obesity: A systematic review and meta-analysis. *Obesity Reviews*. Jul 2021;22e13261. doi:10.1111/obr.13261
27. Wadden TA, Tronieri JS, Butryn ML. Lifestyle Modification Approaches for the Treatment of Obesity in Adults. *American Psychologist*. Feb-Mar 2020;75(2):235-251. doi:10.1037/amp0000517
28. Vieira RFL, Munoz VR, Junqueira RL, et al. Time-restricted feeding combined with aerobic exercise training can prevent weight gain and improve metabolic disorders in mice fed a high-fat diet. *Journal of Physiology-London*. Feb 2022;600(4):797-813. doi:10.1113/jp280820
29. Brady AJ, Langton HM, Mulligan M, Egan B. Effects of 8 wk of 16:8 Time-restricted Eating in Male Middle- and Long-Distance Runners. *Medicine and Science in Sports and Exercise*. Mar 2021;53(3):633-642. doi:10.1249/mss.0000000000002488
30. Moro T, Tinsley G, Longo G, et al. Time-restricted eating effects on performance, immune function, and body composition in elite cyclists: a randomized controlled trial. *Journal of the International Society of Sports Nutrition*. Dec 2020;17(1)65. doi:10.1186/s12970-020-00396-z
31. Tovar AP, Richardson CE, Keim NL, Van Loan MD, Davis BA, Casazza GA. Four Weeks of 16/8 Time Restrictive Feeding in Endurance Trained Male Runners Decreases Fat Mass, without Affecting Exercise Performance. *Nutrients*. Sep 2021;13(9)2941. doi:10.3390/nu13092941
32. Haganes KL, Silva CP, Eyjolfsdottir SK, et al. Time-restricted eating and exercise training improve HbA1c and body composition in women with overweight/obesity: A randomized controlled trial. *Cell Metabolism*. Oct 2022;34(10):1457-+. doi:10.1016/j.cmet.2022.09.003
33. Bhutani S, Klempel MC, Kroeger CM, Trepanowski JF, Varady KA. Alternate day fasting and endurance exercise combine to reduce body weight and favorably alter plasma lipids in obese humans. *Obesity*. 2013;21(7):1370-1379.

Supplementary Note 2

CONSORT 2010 checklist of information to include when reporting a randomised trial*

Section/Topic	Item No	Checklist item	Reported on page No
**Title and abstract**			
	1a	Identification as a randomised trial in the title	Page 1, title
	1b	Structured summary of trial design, methods, results, and conclusions (for specific guidance see CONSORT for abstracts)	Page 1, Abstract
**Introduction**			
Background and objectives	2a	Scientific background and explanation of rationale	Page 2-Page 3
	2b	Specific objectives or hypotheses	Page 3
**Methods**			
Trial design	3a	Description of trial design (such as parallel, factorial) including allocation ratio	Page 10
	3b	Important changes to methods after trial commencement (such as eligibility criteria), with reasons	Page 10-11
Participants	4a	Eligibility criteria for participants	Page 10
	4b	Settings and locations where the data were collected	Page 10
Interventions	5	The interventions for each group with sufficient details to allow replication, including how and when they were actually administered	Page 11-12
Outcomes	6a	Completely defined pre-specified primary and secondary outcome measures, including how and when they were assessed	Page 12-14
	6b	Any changes to trial outcomes after the trial commenced, with reasons	Page 13
Sample size	7a	How sample size was determined	Page 11
	7b	When applicable, explanation of any interim analyses and stopping guidelines	NA
Randomisation:			
Sequence generation	8a	Method used to generate the random allocation sequence	Page 10
	8b	Type of randomisation; details of any restriction (such as blocking and block size)	Page 10
Allocation concealment mechanism	9	Mechanism used to implement the random allocation sequence (such as sequentially numbered containers), describing any steps taken to conceal the sequence until interventions were assigned	Page 10
Implementation	10	Who generated the random allocation sequence, who enrolled participants, and who assigned participants to interventions	Page 10
Blinding	11a	If done, who was blinded after assignment to interventions (for example, participants, care providers, those assessing outcomes) and how	Page 10

Section/Topic	Item No	Checklist item	Reported on page No
	11b	If relevant, description of the similarity of interventions	NA
Statistical methods	12a	Statistical methods used to compare groups for primary and secondary outcomes	Page 14
	12b	Methods for additional analyses, such as subgroup analyses and adjusted analyses	Page 14
**Results**			
Participant flow (a diagram is strongly recommended)	13a	For each group, the numbers of participants who were randomly assigned, received intended treatment, and were analysed for the primary outcome	Page 4, Figure 1
	13b	For each group, losses and exclusions after randomisation, together with reasons	Page 4, Figure 1
Recruitment	14a	Dates defining the periods of recruitment and follow-up	Page 10
	14b	Why the trial ended or was stopped	NA
Baseline data	15	A table showing baseline demographic and clinical characteristics for each group	Page 4, Table 1
Numbers analysed	16	For each group, number of participants (denominator) included in each analysis and whether the analysis was by original assigned groups	Page 4, 14, Figure 1
Outcomes and estimation	17a	For each primary and secondary outcome, results for each group, and the estimated effect size and its precision (such as 95% confidence interval)	Page 4-6
	17b	For binary outcomes, presentation of both absolute and relative effect sizes is recommended	NA
Ancillary analyses	18	Results of any other analyses performed, including subgroup analyses and adjusted analyses, distinguishing pre-specified from exploratory	Page 6
Harms	19	All important harms or unintended effects in each group (for specific guidance see CONSORT for harms)	Page 6
**Discussion**			
Limitations	20	Trial limitations, addressing sources of potential bias, imprecision, and, if relevant, multiplicity of analyses	Page 9
Generalisability	21	Generalisability (external validity, applicability) of the trial findings	Page 9-10
Interpretation	22	Interpretation consistent with results, balancing benefits and harms, and considering other relevant evidence	Page 7-10
**Other information**			
Registration	23	Registration number and name of trial registry	Page 10, Abstract
Protocol	24	Where the full trial protocol can be accessed, if available	Supplementary Note 1
Funding	25	Sources of funding and other support (such as supply of drugs), role of funders	Page 19

Citation: Schulz KF, Altman DG, Moher D, for the CONSORT Group. CONSORT 2010 Statement: updated guidelines for reporting parallel group randomised trials. BMC Medicine. 2010;8:18. © 2010 Schulz et al. This is an Open Access article distributed under the terms of the Creative Commons Attribution License (http://creativecommons.org/licenses/by/2.0), which permits unrestricted use, distribution, and reproduction in any medium, provided the original work is properly cited.

*We strongly recommend reading this statement in conjunction with the CONSORT 2010 Explanation and Elaboration for important clarifications on all the items. If relevant, we also recommend reading CONSORT extensions for cluster randomised trials, non-inferiority and equivalence trials, non-pharmacological treatments, herbal interventions, and pragmatic trials. Additional extensions are forthcoming: for those and for up-to-date references relevant to this checklist, see www.consort-statement.org.
