# Supplementary material for: Flexible time-restricted eating combined with exercise in a free-living setting for middle-aged women with overweight/obesity: a randomized controlled trial
Source: Nat Commun. 2025 Nov 27;16:10659. doi: 10.1038/s41467-025-65678-z (PMC12660762; doi:10.1038/s41467-025-65678-z)
Supplement: Supplementary file 2 — Reporting Summary [file 41467_2025_65678_MOESM2_ESM.pdf]

# nature portfolio

Corresponding author(s): Stephen Heung-sang Wong

Last updated by author(s): Sep 10, 2025

# Reporting Summary

Nature Portfolio wishes to improve the reproducibility of the work that we publish. This form provides structure for consistency and transparency in reporting. For further information on Nature Portfolio policies, see our Editorial Policies and the Editorial Policy Checklist.

## Statistics

For all statistical analyses, confirm that the following items are present in the figure legend, table legend, main text, or Methods section.

| n/a | Confirmed | |
|---|---|---|
| ☐ | ☒ | The exact sample size (*n*) for each experimental group/condition, given as a discrete number and unit of measurement |
| ☐ | ☒ | A statement on whether measurements were taken from distinct samples or whether the same sample was measured repeatedly |
| ☐ | ☒ | The statistical test(s) used AND whether they are one- or two-sided<br>*Only common tests should be described solely by name; describe more complex techniques in the Methods section.* |
| ☐ | ☒ | A description of all covariates tested |
| ☐ | ☒ | A description of any assumptions or corrections, such as tests of normality and adjustment for multiple comparisons |
| ☐ | ☒ | A full description of the statistical parameters including central tendency (e.g. means) or other basic estimates (e.g. regression coefficient) AND variation (e.g. standard deviation) or associated estimates of uncertainty (e.g. confidence intervals) |
| ☐ | ☒ | For null hypothesis testing, the test statistic (e.g. *F*, *t*, *r*) with confidence intervals, effect sizes, degrees of freedom and *P* value noted<br>*Give P values as exact values whenever suitable.* |
| ☒ | ☐ | For Bayesian analysis, information on the choice of priors and Markov chain Monte Carlo settings |
| ☐ | ☒ | For hierarchical and complex designs, identification of the appropriate level for tests and full reporting of outcomes |
| ☒ | ☐ | Estimates of effect sizes (e.g. Cohen's *d*, Pearson's *r*), indicating how they were calculated |

*Our web collection on statistics for biologists contains articles on many of the points above.*

## Software and code

Policy information about availability of computer code

| | |
|---|---|
| Data collection | Microsoft excel (Microsoft 365 Version 2108) |
| Data analysis | SPSS statistics, version 28.0 (IBM Corp., Armonk, NY, USA); Figure design: GraphPad Prism 9.0 (Graphpad software, La Jolla, CA, USA) |

For manuscripts utilizing custom algorithms or software that are central to the research but not yet described in published literature, software must be made available to editors and reviewers. We strongly encourage code deposition in a community repository (e.g. GitHub). See the Nature Portfolio guidelines for submitting code & software for further information.

## Data

Policy information about availability of data

All manuscripts must include a data availability statement. This statement should provide the following information, where applicable:

- Accession codes, unique identifiers, or web links for publicly available datasets
- A description of any restrictions on data availability
- For clinical datasets or third party data, please ensure that the statement adheres to our policy

All data supporting the findings of this study are included in the main manuscript and its supplementary information files. Additional de-identified participant data are not publicly available due to confidentiality restrictions. However, these data can be provided by the corresponding author upon reasonable request for academic purposes.

# Research involving human participants, their data, or biological material

Policy information about studies with human participants or human data. See also policy information about sex, gender (identity/presentation), and sexual orientation and race, ethnicity and racism.

| | |
|---|---|
| Reporting on sex and gender | Only female participants were involved in this study |
| Reporting on race, ethnicity, or other socially relevant groupings | We included Chinese females aged 40-60 years old, who are overweight/obesity. |
| Population characteristics | The baseline population characteristics were listed in Table 1.<br>Inclusion criteria: 1) Chinese females aged 40-60 years old; 2) Obesity/overweight: body mass index ≥ 23kg/m2; 3) physically inactive: ≤ 150 minutes/week of moderate- to vigorous-intensity physical activity; 4) Weight stable for 3 months prior to the beginning of the study (gain or loss < 4kg); 5) Baseline eating period is ≥ 10 h per day.<br><br>The exclusion criteria of participants include 1) Known cardiovascular disease; 2) Type 1 or 2 diabetes mellitus; 3) Currently taking medications that could affect study outcomes. For example, antihypertension medication and taking glucose-lowering or lipid-lowering medication; 4) Known musculoskeletal disease or injury that will affect the exercise; 5) Night shift workers; 6) Breastfeeding, pregnant or trying to become pregnant; 7) Smokers; 8) Currently on a special or prescribed diet for other reasons. |
| Recruitment | Participants were recruited from the general community through various means, including advertisement via posters on campus and social media. |
| Ethics oversight | Ethical approval was obtained from the Joint Chinese University of Hong Kong-New Territories East Cluster Clinical Research Ethics Committee (reference number 2023.238-T), and the study was registered at the Chinese Clinical Trial Registry (ChiCTR2300074846). |

Note that full information on the approval of the study protocol must also be provided in the manuscript.

# Field-specific reporting

Please select the one below that is the best fit for your research. If you are not sure, read the appropriate sections before making your selection.

☐ Life sciences ☒ Behavioural & social sciences ☐ Ecological, evolutionary & environmental sciences

For a reference copy of the document with all sections, see nature.com/documents/nr-reporting-summary-flat.pdf

# Behavioural & social sciences study design

All studies must disclose on these points even when the disclosure is negative.

| | |
|---|---|
| Study description | This single-center, parallel-group, assessor-blinded, four-arm randomized controlled trial was conducted at a single research site in Hong Kong to investigate the isolated and combined effects of 12 weeks of flexible time restricted eating (flexTRE) and aerobic exercise (EX) compared with a non-intervention control group (CON) in a free-living setting on body composition and metabolic health in middle-aged women with overweight and obesity. The study is a quantitative study. |
| Research sample | A total of 104 eligible volunteers were enrolled in the trial, randomized at a 1:1:1:1 ratio into the flexTRE, EX, flexTRE combined with EX (flexTRE+EX), and CON, each comprising 26 individuals. Ninety-eight participants (94.2%) completed the full 12-week trial duration. The study sample consisted of 104 middle-aged females participants, with a mean age of 48 years old. All participants identified as female. |
| Sampling strategy | Participants were recruited from the general community through advertisements placed on campus and social media. The sample is homogenous in terms of age and sex, which was intentional to meet the study's objectives. The sampling strategy for this study is voluntary sampling. The sample likely represents individuals with higher health literacy, access to social media, and proximity to a university community. Therefore, the generalizability of the findings may be somewhat limited to populations with similar characteristics.<br><br>Sample size estimation was conducted using G*Power software based on an effect size derived from a previous study examining the combined effects of ADF with endurance exercise in adults with obesity. Based on these data, an effect size for the group-by-time interaction was calculated (Effect size f = 0.18). Using this effect size, a power analysis determined that 23 participants per group would be required to achieve 80% statistical power at a two-sided significance level of α = 0.05. To account for an anticipated dropout rate of 10%, the study aimed to enroll a total of at least 102 participants, with 26 participants allocated to each of the four groups. |
| Data collection | Pre- and post-intervention evaluations were conducted to assess outcome measures. Outcome assessments were performed according to standardized procedures. Trained research personnel conducting the assessments were blinded to the intervention |

| | |
|---|---|
| | allocation. The primary outcome was fat mass. Secondary outcomes include the other body composition parameters such as body mass, body fat percentage, fat-free mass, BMI, waist and hip circumferences, waist-hip ratio and outcomes encompassed biomarkers associated with metabolic health, comprising fasting glucose, HbA1c, insulin, HOMA-IR, QUICKI, TC, TG, HDL, LDL, leptin, and adiponectin, and resistin. Blood pressure and resting heart rate measurements were also recorded. Additionally, subjective questionnaires were utilized to evaluate the quality of life, sleep quality, and mood profile. |
| Timing | Participant enrollment began in September 2023, with post-assessment concluding in July 2024. |
| Data exclusions | We conducted intention-to-treat (ITT) and Per-protocol (PP) analyses. The ITT analysis included all randomized participants, which reflects the intervention's real-world applicability. In contrast, the PP analysis included only those participants who completed all baseline and post-intervention assessments. |
| Non-participation | A total of 98 participants completed the 12-week intervention. Attrition rates (94.2%) were as follows: two participants in flexTRE (one lost to contact, one because of time constraint), two in EX (one lost to contact, one because of time constraint), one in flexTRE +EX (because of time constraint), and one in CON (not satisfied with the allocation) did not complete the trial. |
| Randomization | Those meeting the inclusion criteria were provided with detailed study information and gave written informed consent voluntarily before enrollment. Eligible participants were randomly assigned to one of four groups in a 1:1:1:1 ratio using an online random number generator with a block size of 8, conducted by an independent researcher. Group assignments were disclosed to participants only after they completed all baseline assessments, using sealed envelopes to ensure confidentiality. Outcome assessors were blinded to group allocation to minimize bias. |

# Reporting for specific materials, systems and methods

We require information from authors about some types of materials, experimental systems and methods used in many studies. Here, indicate whether each material, system or method listed is relevant to your study. If you are not sure if a list item applies to your research, read the appropriate section before selecting a response.

## Materials & experimental systems

| n/a | Involved in the study |
|---|---|
| ☒ | ☐ Antibodies |
| ☒ | ☐ Eukaryotic cell lines |
| ☒ | ☐ Palaeontology and archaeology |
| ☒ | ☐ Animals and other organisms |
| ☐ | ☒ Clinical data |
| ☒ | ☐ Dual use research of concern |
| ☒ | ☐ Plants |

## Methods

| n/a | Involved in the study |
|---|---|
| ☒ | ☐ ChIP-seq |
| ☒ | ☐ Flow cytometry |
| ☒ | ☐ MRI-based neuroimaging |

# Clinical data

Policy information about clinical studies

All manuscripts should comply with the ICMJE guidelines for publication of clinical research and a completed CONSORT checklist must be included with all submissions.

| | |
|---|---|
| Clinical trial registration | Ethical approval was obtained from the Joint Chinese University of Hong Kong-New Territories East Cluster Clinical Research Ethics Committee (reference number 2023.238-T), and the study was registered at the Chinese Clinical Trial Registry (ChiCTR2300074846). |
| Study protocol | Submitted with the manuscript. |
| Data collection | This is a single-center, parallel-group, assessor-blinded, four-arm randomized controlled trial. Participant enrollment began in September 2023, with post-assessment concluding in July 2024 at a laboratory of the Department of Sports Science and Physical Education in the Chinese University of Hong Kong. |
| Outcomes | The primary outcome was fat mass which was evaluated following an 8-hour fast, with participants refraining from consuming any liquids, using a bioelectrical impedance analyzer (MC-780 MA, Tanita, Japan) that has been previously validated compared to dual-energy x-ray absorptiometry [fat mass (kg) ICC: 0.925]. Measurements were conducted following a standardized protocol at the same time each morning. Participants wore light, form-fitting attire and remained barefoot, with 0.5 kg consistently deducted to account for their clothing. Prior to each measurement, participants' date of birth, gender, and height were entered into the analyzer. The "normal" body type setting (rather than "athlete") and the Asian judgment setting were applied. Body mass index (BMI) was calculated according to the World Health Organization (WHO) guidelines, ensuring a consistency and standardized assessment across all tests.<br><br>Secondary outcomes include the other body composition parameters such as body mass, body fat percentage, fat-free mass, BMI, waist and hip circumferences, waist-hip ratio and outcomes encompassed biomarkers associated with metabolic health, comprising fasting glucose, HbA1c, insulin, HOMA-IR, QUICKI, TC, TG, HDL, LDL, leptin, and adiponectin, and resistin. Blood pressure and resting heart rate measurements were also recorded. Additionally, subjective questionnaires were utilized to evaluate the quality of life, sleep quality, and mood profile. Details could be found in the method part. |

## Plants

| | |
|---|---|
| Seed stocks | *Report on the source of all seed stocks or other plant material used. If applicable, state the seed stock centre and catalogue number. If plant specimens were collected from the field, describe the collection location, date and sampling procedures.* |
| Novel plant genotypes | *Describe the methods by which all novel plant genotypes were produced. This includes those generated by transgenic approaches, gene editing, chemical/radiation-based mutagenesis and hybridization. For transgenic lines, describe the transformation method, the number of independent lines analyzed and the generation upon which experiments were performed. For gene-edited lines, describe the editor used, the endogenous sequence targeted for editing, the targeting guide RNA sequence (if applicable) and how the editor was applied.* |
| Authentication | *Describe any authentication procedures for each seed stock used or novel genotype generated. Describe any experiments used to assess the effect of a mutation and, where applicable, how potential secondary effects (e.g. second site T-DNA insertions, mosiacism, off-target gene editing) were examined.* |
